# Performance of Capsules in Self-Healing Cementitious Material

**DOI:** 10.3390/ma15207302

**Published:** 2022-10-19

**Authors:** Mouna A. Reda, Samir E. Chidiac

**Affiliations:** Department of Civil Engineering, McMaster University, 1280 Main Street West, Hamilton, ON L8S 4L7, Canada

**Keywords:** cement, self healing, capsules, FE model, compatibility

## Abstract

Encapsulation is a very promising technique that is being explored to enhance the autonomous self-healing of cementitious materials. However, its success requires the survival of self-healing capsules during mixing and placing conditions, while still trigger the release of a healing agent upon concrete cracking. A review of the literature revealed discontinuities and inconsistencies in the design and performance evaluation of self-healing cementitious material. A finite element model was developed to study the compatibility requirements for the capsule and the cementing material properties while the cement undergoes volume change due to hydration and/or drying. The FE results have provided insights into the observed inconsistencies and the importance of having capsules’ mechanical and geometrical properties compatible with the cementitious matrix.

## 1. Introduction

Hydraulic cement shrinks when it chemically reacts with water to form calcium silicate hydrate (C-S-H), the glue that binds aggregates to form concrete. Early-age cracks, caused by chemical reactions, drying, and/or temperature fluctuations, vary in crack opening displacement (COD) between 10 and 100 μm [1,2]. Although fine cracks with COD less than 200–300 μm are considered too small to affect the performance and durability of concrete [3,4,5,6], many studies observed that COD between 50 and 200 μm can affect the water permeability of concrete [7,8,9,10]. Accordingly, COD greater than and/or equal to 50 μm can allow entry of water and deleterious liquids to the concrete core, and thus facilitates the occurrences of concrete’s chemical, physical, and/or electrochemical deterioration mechanisms. The results have been a deteriorated concrete infrastructure with an estimated repair or replacement cost between USD 18 and USD 21 billion in the United States alone [11,12]. Hence, autonomous healing, which can be achieved by adding cementing materials, microorganisms, or other healing agents that react chemically with the cementitious matrix, has been suggested and studied as a potential remedy [13,14,15,16,17,18,19,20,21,22,23,24,25,26,27,28]. The encapsulation of the healing agents has been used to protect microorganisms from the harsh conditions during mixing and cement hydration, and to protect cementitious and polymeric materials from early activation [29,30,31,32,33,34,35,36,37,38,39,40,41,42,43,44,45,46,47,48,49,50,51,52,53,54,55]. The capsules, which are in most cases spherical in shape and range from μm to mm [56], can be effective in sealing and/or healing cracked concrete, provided they are uniformly distributed [41] and are bonded to the cement paste, and the crack opening is limited to 200 μm [20].

Healing occurs when the capsule ruptures upon intercepting a propagating crack, and the healing agent bonds the cracked surfaces. Depending on the mechanical properties and bond strength of the shell, the capsule can break during concrete mixing or placement and thus spoiling the healing agent or can debond instead of rupturing upon intercepting a crack. Accordingly, the healing effectiveness of the capsules in a cementitious matrix depends equally on the distribution and volume fraction of capsules, and on the mechanical properties, fracture energy, and interaction between the capsule and the cementitious matrix. This wicked problem is further challenged by the absence of standardized test methods for determining the performance of self-healing cementitious systems and the capsules. Consequently, the performance of self-healing cementitious systems using capsules, reported in the scientific literature, has been inconsistent [57,58,59,60,61]. 

Healing of a cracked young cementitious material poses additional challenges due to the evolution of microstructure as cement hydrates. Moreover, young cementitious material is most susceptible to cracking and these early-age cracks are the root cause of most concrete deterioration in civil infrastructure. This study, which is numerical in scope, aims to investigate the effectiveness of capsules in healing cracked young concrete and identify some of the design requirements necessary for an effective self-healing cementitious system. The paper includes a brief review of the relevant experimental and analytical studies reported in the literature, methodology developed for this investigation, analysis, and model results. Discussion of the results, which includes a comparison to the reported literature results, is subsequently presented.

## 2. Performance of Self-Healing Cementitious System

### 2.1. Experimental Studies

#### 2.1.1. Capsule

The capsules in self-healing cementitious system which act as carriers for the healing agent, release the healing agent when mechanically triggered. As such, several shell materials with different healing agents have been studied, as documented in Table 1. The role of the shell, which encapsulates the healing agent, is to protect the agent from rupturing during concrete mixing and placing, and yet cracks and facilitates the release of the agent upon concrete cracking. In brief, the capsule’s mechanical properties and geometry are pivotal for the success of self-healing cementitious systems. Although several test methods have been employed to measure the mechanical properties of the capsules, as summarized in Table 2, there are no standard test method nor guidance on the target values for capsule properties in self-healing cementitious systems.

The single-microcapsule technique was employed by Liu et al. [78] to measure the compressive displacement and corresponding force of Poly-Urethane (PU) capsules using micro-upsetting instruments. The single capsule started to burst when the ratio of compressed displacement to initial diameter reached 60%. The same technique was also used by Keller and Sottos [77] to evaluate the mechanical properties of dry and immersed DiCycloPentaDiene (DCPD)-filled Urea-Formaldehyde (UF) capsules. The failure, which occurred when the displacement reached 40% of the initial diameter, was due to leaked DCPD. They reported that the capsule did not burst or buckle and attributed the failure to localized damage due to the large radius of curvature. The corresponding mechanical properties were deduced from the measured load-displacement curve and membrane theory model assuming isotropic nonlinear-elastic [78] and isotropic linear-elastic material [77], respectively.

Lee et al. [73] adopted a nanoindentation technique to measure the micromechanical properties of an epoxy-filled Poly-Melamine-Formaldehyde (PMF) capsule. Using the measured load-displacement curve, the hardness and elastic modulus of the capsule were calculated. This experimental study is documented for material reference as the capsules were intended for self-healing polymers. Nanoindentation was also employed by Lv et al. [32] to measure the elastic modulus and rupture force of DCPD-filled Phenol-Formaldehyde (PF) capsules. The elastic modulus was deduced from the linear phase of the load-displacement curve at a displacement between 600 and 900 nm. The rupture force was determined at a depth of 5 μm. The same technique was also used by Lv et al. [79] to determine the elastic modulus and hardness of the shell-cement paste interface. To simulate the interface between cement paste and PF capsules, a small piece of the shell material resin was placed on top of the cement paste sample and sealed by epoxy resin. The mechanical properties of the shell material, cement paste, and interface were measured after curing for 28 d. The elastic moduli for the cement paste, shell material, and the interface, were found to be 16 GPa, 5.5 GPa, and 4.75 GPa, respectively, and the corresponding tensile strengths were 3 MPa, 1.10 MPa, and 0.12 MPa.

Studies that were undertaken to study the performance of self-healing cementitious systems, also tested the properties of General-Purpose Polystyrene (GPPS), Acrylonitrile-Butadiene-Styrene (ABS), and High Impact-resistance Polystyrene (HIPS) capsules using compression test [80]. The test aimed to mimic the interaction between the capsules and cementitious materials. They reported that the measured load-displacement curves are not sufficient for determining the capsules’ material properties as further analyses are needed to account for the effects of capsule bending resistance. This review, although brief, shows the challenges in determining the properties of the capsules and the interface between the capsules and cement paste. The results do, however, confirm that the shell elastic modulus and rupture stress depend on the capsule material type and geometric properties, as well as on the testing method and mechanics theory adopted to estimate the values.

The success of capsules in self-healing systems also depends on their survivability rate during harsh concrete mixing and placing conditions, and on their ability to withstand the high alkaline nature of cementitious systems. A search of the literature revealed that the survival of the capsules was deduced from visual inspection using scanning electron microscopy (SEM) images of pre-cracked hardened concrete samples [65]. Although this approach provides an indirect measurement of the capsule’s survivability rate, the results are yet to be proven statistically as representative of the whole sample. The SEM images before and after mixing were compared, and it was observed that the capsules survived the mixing, and they have a good bond with the cement paste matrix. Furthermore, Hilloulin et al. [36] and Lv et al. [34] tested the effects of alkaline environment on polymeric tubes and PF capsules, respectively. The former employed cement slurry with pH ~ 12.5–13 for a period of 7 and 14 d and measured the effect by comparing the tubes’ tensile strength, whereas the latter used Ca(OH)_2_ aqueous solution with a pH ~ 13 for 2 d and visually inspect the capsules using SEM. The results detailed by Hilloulin et al. [36] revealed that the Poly(Lactic Acid) “PLA” and PS tubes did not experience change in their tensile strength after being exposed to an alkaline environment, whereas the Poly(Methyl Methacrylate/n-Butyl MethAcrylate) “P(MMA/n-BMA)” tubes had a lower strength. On the other hand, Lv et al. [34] compared the SEM images for the PF tubes, and no change was observed in the shape after exposing the capsules to an alkaline environment. Although both were attempting to measure the shell chemical resistance during the cement hydration period, their approach differs significantly both in terms of exposure condition and evaluation method. The use of different test methods will automatically yield different results and inconsistencies in performance.

A primary role of the capsules within a self-healing system is to rupture and not debond upon concrete cracking. The former ensures the delivery of the healing agent, whereas the latter results in a loss in crack healing potential. Mechanical properties of cementitious material, capsule, and interfacial zone dictate to some extent the performance of a self-healing system [19,32,79]. Using SEM images of a mechanically triggered crack by means of a compression test, Wang et al. [39] observed that some UF capsules ruptured, whereas others debonded from the 49 MPa compressive strength mortar, but did not explain what caused the difference in performance. Also using the compression test and SEM images, Dong et al. [65] investigated the fractured surfaces of UF capsules. The images revealed that the capsules ruptured with the shell still bonded to the 55.8 MPa compressive strength cement paste. Lv et al. [34] used optical microscope (OM) and X-ray computed tomography (XCT) scanning technology to investigate the fractured surface of cement paste samples cured for 28 d and tested by a three-point bending test. It was observed that some of the capsules were ruptured by the crack, whereas the others were tightly embedded in the matrix indicating a good bond with the matrix. The study acknowledged the weak bond between the microcapsules and the matrix that needs improvement, but without providing guidance. These results not only loosely and qualitatively document the performance of the capsules in cementitious matrix, but also showed that different test methods are used to evaluate the interface between the capsules and the cementitious matrix.

In summary, the capsules were tested to determine if the shell possesses the properties necessary to survive the mixing and placing of the cementitious mixture, and to crack when the cementitious matrix cracks. A range of properties has been reported for the shell’s elastic modulus and rupture stress. Assuming that the test methods are repeatable and consistent, what values should the shell possess so that it can be effective in a self-healing cementitious system? Evidentially, a need exists for developing standard test methods for measuring the geometrical and mechanical properties, durability of the capsule shell, and the properties of the interfacial zone between the capsules and matrix, as well as for establishing the corresponding values that are deemed acceptable for the self-healing cementitious system.

#### 2.1.2. Healing System

The efficiency of a healing system is determined by the release of the healing agent and the healing of the cracks. The former is controlled by the mechanical properties of the capsules, the matrix, and their interfacial zone, whereas the latter requires that the agent flows, fills the volume before hardening, and bonds the faces of the crack. Evaluating the efficiency of healing systems is therefore complex as their performance relies on the congruent occurrence of many events. As such, indirect test methods, such as visual inspection [37,41,81], and measuring the recovery of the mechanical properties [32,33,39,40,42,53,65,71] and/or water/air tightness of the matrix [39,40,42,53,65,66], have been employed to evaluate the performance of the healing system. It is evident that the absence of a standard test method and/or metric, and the use of different test methods have led to the documented inconsistencies in the performance measurement as reported in Table 3. The absence of a metric and/or guideline that prescribe acceptable range of shell material properties, capsule size and concentration, and healing agent properties, causes uncertainties in the healing system performance and can potentially impede its development.

The data in Table 3, which present a representative sample of proposed healing systems and performance test methods, not only reveal their diversity but also show the inconsistencies in the design of the healing system specifically the size and content of the capsules and their compatibility to the cementitious mixture composition. Nonetheless, the following observations have been deduced:

i. Capsules formed using UF are found to range between 10 to 1000 mm in diameter, 0.2 to 8 mm in thickness, and 8 to 39 in the ratio of radius to thickness except for the capsules that were used by Gilford et al. [71] whose ratio is 107 to 5000. Both the radius-to-thickness ratio and diameter of the capsules affect their ability to withstand forces, to develop a mechanical bonding, as well as to effectively deliver the healing agent. The spectrum provides little information and thus confidence on what geometrical properties the capsules need to possess for an effective self-healing system.
ii. UF encapsulating epoxy resin [39,40,53,65,66], Dicyclopentadiene (DCPD), Sodium Silicate [71], and Calcium Nitrate Tetrahydrate [41], have been added to the mortar with varying mixture composition and properties. The reported 28-day mortar compressive strength ranges from 28 to 56 MPa, and the flexural strength from 8.4 to 10.6 MPa. The cementitious mix design is seldom documented in these studies and only some studies reported the mechanical properties of the hardened mixture. The ratio of water to cement and cement to sand, the cement content, capsule content, and other additives are found to significantly vary among the documented studies without any rationale to the design.
iii. Capsules contents are found to range between 0.5 and 12% of the cement content. The broad range of the capsule content used in these studies combined with the absence of any rational to designing self-healing system can discourage the concrete construction industry from experimenting with the self-healing system.
iv. UF, MUF, and PF are used for encapsulation, with UF being the most common, and epoxy resin, DCPD, Sodium Silicate, and Calcium Nitrate Tetrahydrate used as healing agents with epoxy being the most common. The diverse chemical composition and properties of the healing agents provide options, but with no justification or guidance on how to select the healing agents.
v. Test methods not only vary in scale from recovery of mechanical properties and transport properties to recovery of matrix microstructure which includes pores size distribution and porosity, but also the varying ages at which matrix was pre-cracked and tested. These variabilities raise many questions: Is there a difference in material response between mechanically and chemically triggered cracks, i.e., between cracks induced by external loads versus those caused by dimensional changes? Does the cementitious material degree of hydration affect the healing efficiency of the system, specifically the capsule bond strength? The aim of these performance tests appears to test the mechanical and/or durability recovery of mature concrete and provide zero measure of the healing performance at an early age when the cementitious system is most vulnerable to cracking.
vi. Healing performance indicators of the systems appear to be all over the place where the following measures have been reported: average recovery rate, recovery rate, healing rate, crack healing ratio, and healing ratio. For reference, the rate is a measure of two unlike units and should not be used to compare two measurements of the same units. Alternatively, the ratio can provide a measure of the healed system to the uncracked system. Moreover, the reported experimental measurements are concerning as without a measure of certainty in the form of standard deviation, there is zero confidence in their measured values.

The previous studies revealed that using capsules in self-healing cementitious systems without understanding the properties required to ensure compatibility with the cementitious matrix, without a defined aim of the self-healing system being sealing or healing, and without a clear definition of efficiency would lead to inconsistencies in the results, perhaps even for the same type of capsules.

### 2.2. Numerical Studies

Several studies proposed analytical/numerical models to investigate the fracture behavior of the capsules in self-healing systems and evaluate the suitability of their mechanical and geometrical properties and their interactions with the cementitious matrix. Gilabert et al. [82] developed a 2D model to investigate the effect of the interfacial bond strength on the stress concentration around a cylindrical capsule embedded in a cracked linear elastic concrete matrix subjected to uniform uniaxial far-field stress. The interface was represented by a linear cohesive zone model. Perfect and imperfect bonds were investigated with different ranges of shell-to-matrix stiffness ratio, capsule thicknesses, and strength ratio. The results revealed that debonding of the capsule is controlled by the strength ratio namely bond strength to far-field stress, geometric ratio, i.e., capsule thickness to radius, and elastic property ratio of capsule to concrete. The effect of interfacial fracture energy on the capsule debonding behavior was also investigated [83]. The results showed that the fracture energy does not influence the initiation of debonding and that fracture energy greater than 0.5 J/m^2^ has no effect on debonding. However, it affects the brittleness of the process of failure. A model of the three-point bending test employing the extended finite element method (XFEM) and cohesive surface techniques (CS) were used to investigate the effect of inserting tubular glass capsules on the overall beam’s strength, and to study the capsule size and interfacial properties vis-a-vie capsule rupture [84]. The results revealed that the bond strength needs to be at least 2 MPa to ensure the rupture of the capsule, and that by increasing the ratio of the capsule thickness to radius to 0.23, the minimum bond strength required to ensure capsule rupture increases to 5 MPa, which is considered high for a polymer–mortar interface. Li et al. [85] developed a finite element model using the XFEM technique and cohesive zone to simulate crack propagation in a matrix and the potential of capsule debonding. They concluded that debonding depends on the strength ratio between the capsule and the interface. The effect of fracture strength at the interfacial transition zone (ITZ) of a circular capsule with different core-shell thicknesses on the rupturing of the capsule was investigated using a 2D numerical model [86]. The crack path was pre-initiated as a zero-thickness cohesive element through the concrete matrix. They concluded that the probability of capsule rupture is highly influenced by the capsule shell thickness, and when the fracture properties of the interface are equal to the mortar matrix, the probability of capsule rupture increases. A follow-up study was undertaken to study the effects of varying the fracture strengths between capsule, mortar, and the interface on the crack initiation and propagation [87]. The results revealed that having similar fracture strength for the capsule and mortar with higher interfacial strength ensure crack propagation through the capsule.

The results from the numerical studies, although limited, confirm the significance of the capsule geometric properties and compatibility between the capsule, mortar, and interface bond strength, on the performance of self-healing cementitious system. Moreover, these results highlight the significance of mortar properties on the self-healing performance, specifically when considering early-age cracking of cementitious material.

## 3. Methodology

An experimental program was developed using factorial design of experiments [88] to numerically investigate the mechanical interactions between the mortar and the capsules as the mortar cracks due to dimensional changes. The geometrical and mechanical properties of the capsule, and the properties of the mortar and the interface between the mortar and the capsules as a function of age and composition were studied. Two mortar mixes, one mix without supplementary cementing materials (SCM) at age 2 and 28 d, and one mix with SCM consisting of 22% ground granulated blast furnace slag (GGBFS) and 8% silica fume (SF) as cement replacement at age 2 d, were considered for this study. The corresponding mortar mixture composition and properties are given in Table 4. The mortar compressive strength was estimated using the model proposed by Chidiac et al. [89], the modulus of elasticity and tensile strength using the models of ACI 318M [90] and Onken and Rostasy [91], and the fracture toughness using the models of Gustafsson [92] and Hillerborg [93]. The values adopted for the shell geometry and material, and for the interface were selected based on the data reported in the literature and reproduced in Table 5. The factorial design, which uses the variables presented in Table 6, led to 96 combinations that were analyzed using the commercial finite element program ABAQUS [94].

### 3.1. Finite Element Model

**Figure 1:**
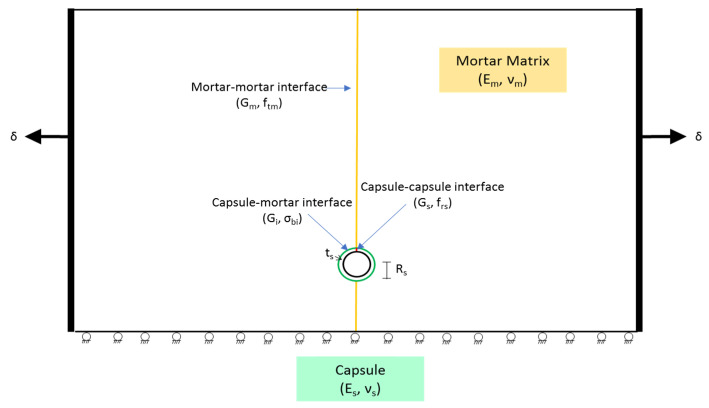
FE model schematic view including geometry, boundary conditions, and loading conditions.

A 2D plane strain finite element model was constructed for this analysis. The idealized model, which is shown in Figure 1, consisted of a 50 mm by 30 mm rectangular-shaped mortar matrix with a single spherical capsule centrally located 25 mm below the top surface, an interface layer around the capsule, and a centrally positioned crack path. Accordingly, three interfaces were considered, mortar-to-mortar interface for where the mortar cracks, capsule-to-mortar interface representing the zone binding the mortar and the capsule, and the capsule-to-capsule interface for where the capsule ruptures. Moreover, two boundary conditions were used, a roller at the bottom surface and a horizontally moving rigid boundary at the two vertical edges of the matrix. The latter simulates the effects of dimensional change, specifically, shrinkage.

**Figure 2:**
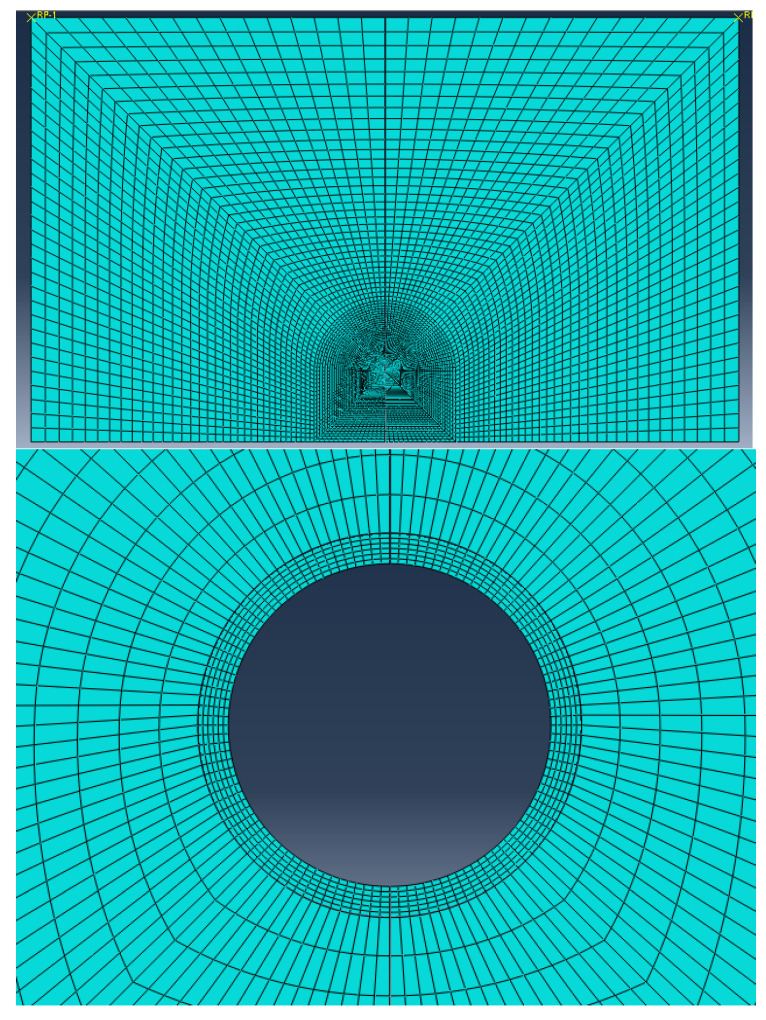
Finite element mesh of the mortar (**top**) and the area around the capsule (**bottom**).

The progressive meshing was used in this study to balance computational efforts and discretization errors. The element size ranged from 2.6 μm around the capsule interface to 1.6 mm at the far edges of the mortar matrix. The generated finite element mesh along with a zoomed-in mesh on the area surrounding the capsule are shown in Figure 2. For reference, 720 and 12,300 CPE8 elements were used to model the capsule and the matrix, respectively.

### 3.2. Damage Model

**Figure 3:**
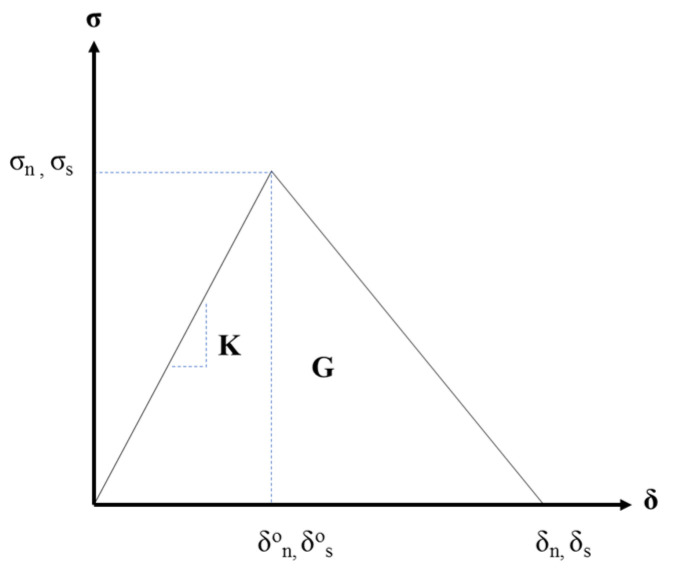
Bilinear traction–separation law [94].

Abaqus cohesive interface surfaces were used to capture the damage as the rigid boundaries move horizontally to simulate the effects of dimensional changes [94]. The elements are used to connect any two surfaces whose separation is governed by a traction-separation law, specifically the matrix–matrix interface, capsule–matrix interface, and capsule–capsule interface, as illustrated in Figure 1. The traction-separation behavior, described in Figure 3, is assumed to be linear elastic until the initiation of damage. Damage is initiated when the stress has reached the bond strength of the capsule–matrix interface, the maximum tensile strength of mortar at the mortar–mortar interface, and the maximum rupture strength of the capsule at the capsule–capsule interface. Thereafter, damage will evolve based on energy dissipation principles and is governed by the interface fracture toughness (G). Moreover, the model assumes that 80% of the capsule surface area is bonded to the surrounding matrix, yielding a 20% reduction in the bond strength between the mortar and the capsule.

## 4. Results, Analyses, and Discussion

**Figure 4:**
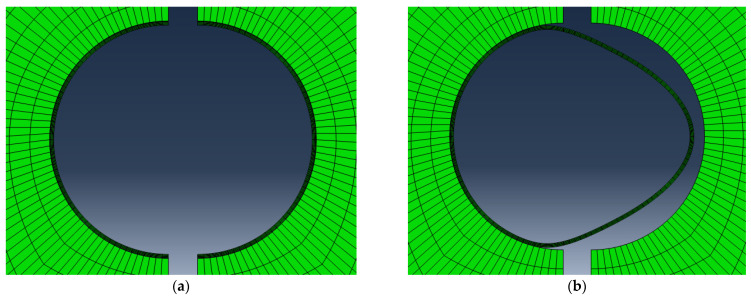
Capsule failure modes: (**a**) rupturing, (**b**) debonding.

**Figure 5:**
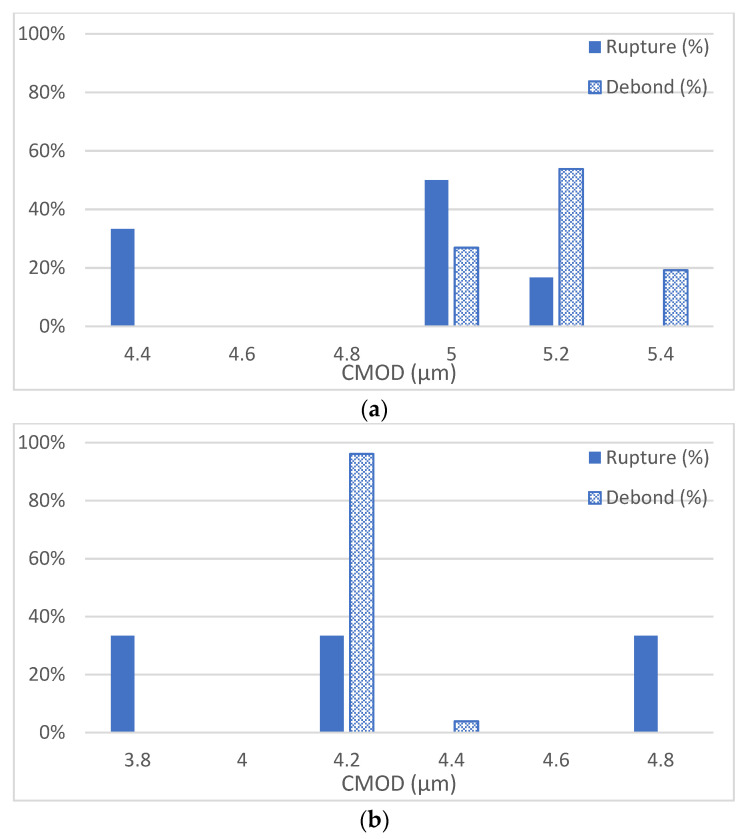

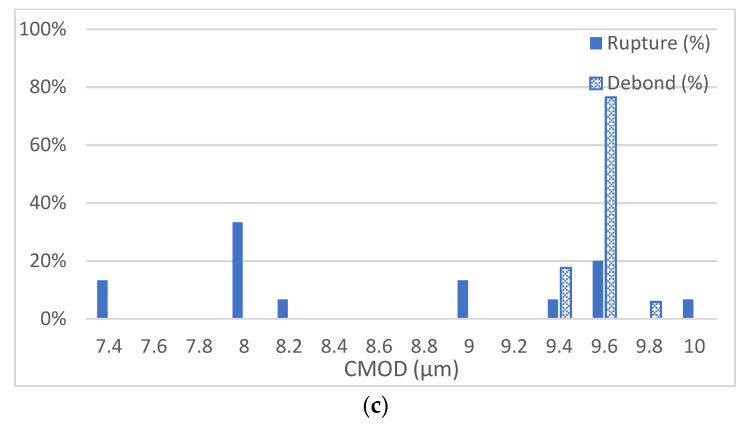
CMOD and capsule failure modes for the three mortar mixes, (**a**) 2 d without SCMs, (**b**) 2 d with SCMs, and (**c**) 28 d without SCMs.

The results from the finite element analyses, in the form of failure mode, and crack mouth opening displacement (CMOD) are reproduced in Table 7. The two failure modes, Rupturing (R) of the shell and Debonding (D) of the capsule, which were captured by the finite element model, are shown in Figure 4. Figure 5 shows the relationship between CMOD and the capsule failure modes for the three mortar mixes. The observed trend clearly indicates that the relationship between the capsule and the mortar is very much influenced by the properties of the mortar. Comparing the mortar mixes without SCM at 2 d and 28 d, the CMOD values are different reflecting the strength development but also the pattern is different. At 2 d, rupturing as the mode of failure is dominant for 33% of the capsules at smaller crack opening but as the crack widens, the mode of failure becomes unpredictable with debonding being more predominant. At 28 d, rupturing as the mode of failure is dominant for 66% of the capsules and is over a broader range. As the crack continues to widen, debonding of the capsule becomes the dominant mode of failure. These results are significant in more than one way, first the age of the mortar or the mechanical property of the mortar dictates the interaction between the capsule and the mortar, and second the size of the crack at 28 d dictates the capsule predominant mode of failure. The latter is significant for the cases where mortar is pre-cracked to study the efficiency of the self-healing system. Comparing the mortar mixes with and without SCM at 2 d, one observes a different pattern. The CMOD values reflect the weaker mortar at 2 d. Moreover, the mode of failure is different for SCM mixes where rupturing is dominant at both ends whereas those without SCM show a transition from one mode to the other. This indicates that the response is very complex for weaker mortar reflecting the early age.

Further examination of the results reveals the interactions between the capsule geometry and property, and the mortar property. For the 2 d mixes without SCMs, and moving from low to high CMOD values, the following observations are deduced: (1) large capsules with thin shell and low rupture strength are for most cases rupturing; (2) for the smaller capsule and thicker shell or high rupture strength, debonding is the predominant mode of failure; (3) there was no dominant failure pattern observed when the capsule rupture strength, the bond strength, and the capsule geometry are found to have equally competing values. The 2 d mixes with SCMs experienced similar behavior but with lower CMOD values, notably: (1) large capsules with thin shell always experienced rupturing despite their rupture strength; (2) capsules with higher rupture strength need large CMOD value to fail without altering the mode of failure being rupture or debond; (3) Small capsules with thicker shell will most likely debond. Moreover, for 28 d mixes without SCMs, the predominant mode of failure is rupturing of the shell at the high CMOD values including thicker shell and smaller capsules except for those with a low capsule radius-to-thickness ratio, specifically 6 to 20. This observation includes the capsules with a very thick shell of 8 μm.

Comparing the model results to the observations made from experimentally reported data on self-healing mortar, one can provide insights into the inconsistencies in the system performance. First, examining the geometry of the capsules it was deduced from the model that most of the capsules whose radius to thickness ranged from 6 to 20 debonded, 20 to 30 debonded or ruptured depending on the capsule rupture strength, and from 33 to 100 ruptured. This implies that the diameter of the capsule, without considering the other geometrical properties such as thickness, and without considering the rupture strength of the capsule is not a sufficient measure to predict the response of the capsule. It should be noted that the capsule used in the model ranged from 100 to 200 μm in diameter and 1 to 8 μm in thickness. Closer examination of Wang et al.’s [39] experimental results reveals that the employed capsules had a radius to thickness ratio ranging from 4 to 38, and that some capsules were ruptured whereas others debonded when tested at 28 d. These experimental observations support the deduced model results where rupturing as the prevalent mode of failure occurs when the ratio is between 33 and 100. Likewise, Lv et al. [34] reported that some of the capsules were ruptured by the crack, whereas others remained intact in the cement paste. Again, their capsules’ radius to thickness ratio ranged from 0.20 to 45. Dong et al. [65] noted that the capsules ruptured when tested but unfortunately made no mention of the capsules debonding or of the capsules thickness. Second, the mechanical properties of the mortar when pre-cracked, which are dictated by the age and composition of the mixture, and the crack width are found to highly affect the capsule mode of failure. These observations further explain the inconsistencies in the performance of self-healing mortar. Third, the absence of a standard test that accounts for the properties of the mortar and capsule, and a clear methodology for pre-cracking and testing performance of self-healing mortar have added to the uncertainty in the body of knowledge.

## 5. Conclusions

The results from the analytical study and the review of the literature yield the following conclusions:

(1) There is a need for developing standard test methods to measure the capsules geometry, being diameter and thickness, and mechanical properties, and the mechanical properties of the interface between the mortar and the capsule.
(2) There is a need for developing standard test methods for measuring the survival rate of capsules during mixing and placing of concrete as a pre-requisite to determining efficiency of the self-healing cementitious system.
(3) There is no clear definition of self-healing efficiency nor a define method for measuring self-healing efficiency of mortar and other cementitious systems.
(4) Inconsistencies in the reported self-healing mortar performance are attributed to the inter-relationship between the geometry of the capsules, the properties of the capsules, the properties of the mortar, and the pre-crack width induced in the mortar.
(5) The capsules’ radius to thickness is found to significantly affect the capsule mode of failure.
(6) The crack opening affects the capsule failure mode differently depending on the age and composition of the mortar, and properties of the capsule.
(7) The age of the mortar is important when testing the self-healing system, especially when mortar is susceptible to cracking at early age. It is pivotal to check the status of the capsules due to early-age cracking before moving forward with measuring the efficiency of the self-healing system.

## Figures and Tables

**Table 1:** Common shell materials and healing agents used in self-healing concrete.

Shell Material	Healing Agent	Reference
Perspex	Epoxy Resin	[62]
Ceramic	Polyurethane (PU)	[17]
	Methyl Methacrylate (MMA)	[29]
Glass	Epoxy Resin	[62]
	Methyl Methacrylate (MMA)	[25]
	Cyanoacrylates (CA)	[26]
Borosilicate glass	Methyl Methacrylate (MMA)	[29]
	Polyurethane (PU)	[17,38,63]
	Cyanoacrylates (CA)	[15,16]
Quartz glass	Polyurethane (PU)	[50]
Urea-formaldehyde (UF)	Epoxy Resin	[39,40,53,64,65,66]
	Dicyclopentadiene (DCPD)	[67,68,69,70,71]
Melamine Urea-formaldehyde (MUF)	Epoxy Resin	[33]
	Dicyclopentadiene (DCPD)	[72]
	Epoxy Resin	[73]
Phenol-formaldehyde (PF)	Dicyclopentadiene (DCPD)	[32,34]
	Epoxy Resin	[74]
	Dicyclopentadiene (DCPD)	[75,76,77]
Polystyrene (PS)	Methyl Methacrylate (MMA)	[42]
	Polyurethane (PU)	[36]

**Table 2:** Properties of capsules extracted from the literature.

Shell Material [Test Method]	Average Size (D) (µm)	Shell Thickness (t) (µm)	Elastic Modulus (GPa)	Bursting/Rupture Stress (MPa)	Reference
Poly-Urethane (PU)	50–100	1–2	0.0029	0.026	[78]
Urea-Formaldehyde (UF)	65 ± 7 dry	0.175 ± 0.033	3.7 ± 0.5	0.8 ± 0.3	[77]
	187 ± 15 dry		3.6 ± 0.4	0.24 ± 0.04	
	213 ± 12 immersed		3.9 ± 0.7	0.14 ± 0.02	
Poly-Melamine-Formaldehyde (PMF)	10–150	0.2	4.66	-	[73]
Phenol-Formaldehyde (PF)	50–200	29.96	2.2 ± 0.8	68.5 ± 41.6	[32]
	200–400			96.8 ± 23.5	
	400–600			198.5± 31.6 mN	

**Table 3:** Performance of self-healing system in concrete applications.

Healing Agent	Shell Material	Performance Criteria	Capsule Size (μm)	Capsule Content (%)	Pre-Loading Condition	Curing Conditions and Testing Age	Testing Methodology	Reference
Epoxy resin	UF	Mechanical properties and durability	73–309	3, 6, 9	30%, 50%, 70% of maximum compressive/flexural strength	Pre-cracked after curing for 28 d (RH > 90%, 20 °C), then left to heal for 3 d (same curing conditions)	- Compressive strength test - Three-point bending test - RCM test	[39]
Epoxy resin	UF	Mechanical properties and durability	45–185	3, 6, 9	60% of maximum compressive strength	Pre-cracked after curing for 28 d (RH > 90%, 20 °C), then left to heal for 7 d (cured in a box at temp. < 50 °C)	- Compressive strength test - DMA test - MIP test	[53]
Epoxy resin	UF	Mechanical properties and durability	132, 180, 230	2, 4, 6, 8	60% of maximum compressive strength	-	- Compressive strength test - RCM test	[40]
Epoxy resin	UF	Mechanical properties and durability	132, 180, 230	2, 4, 6, 8	30–70% of maximum compressive strength	Cured for 60 d in the curing chamber (95 ± 5% RH, 20 ± 2 °C), then pre-cracked and left to heal at a temperature of 30–60 °C for 3 d, 5 d, 7 d, 14 d and 28 d	- Compressive strength test - RCM test - MIP test	[65,66]
DCPD and Sodium Silicate	UF	Mechanical properties	75–1000	0.5, 1.0, 2.5, 5.0 (Sodium Silicate), 0.25 (DCPD)	70% of maximum compressive strength.	Steam cured for 7 d at 20–25 °C, then reloaded three cycles before left to heal in curing room for 48 h	- Compressive strength test	[71]
Calcium Nitrate Tetrahydrate	UF	Visual and crack width	22–59	0.5, 0.75	Flexural damage up to sudden change in the displacement	Cured for 28 d (95% RH), oven-dried for 3 d (60 °C), then pre-cracked, water immersed for 7, 21, 42 d, and oven-dried again for 3 d (60 °C)	- ESEM/EDS	[41]
Sodium Silicate	Double-walled PU/UF	Visual and crack depth	-	2.5, 5	Flexural damage up to load of 500 kg	Cured water for 7 d, pre-cracked then left to heal for 2 weeks	- PUNDIT device	[37]
Epoxy resin	MUF	Mechanical properties	10–1800	1, 2, 4	30, 60, 80% of maximum load resistance	Cured for 28 d (≥95% RH, 20 ± 2 °C), left to heal for 2 h after pre-cracked, then tested up to failure	- Three-point bending test	[33]
DCPD	PF	Mechanical properties	50–600	4–12	Loaded up to failure	Cured for 28 d in wet chamber (25 °C, 95% RH)	- Compressive strength test	[32]
MMA	PS	Mechanical properties and durability	4.15	1.5	80% of maximum compressive strength	Cured in wet chamber for 28 d (≥95% RH, 20 ± 2 °C. Samples of 1 d and 28 d are pre-cracked, rest for 24 h, then cured for another 24 h in vacuum-dried room (for permeability)/subjected to cyclically loading between 25–95% of maximum compressive strength (for fatigue)	- Gas permeability test using liquid methanol - Fatigue test under uniaxial compression cyclic loading	[42]
CSA	PS	Visual and crack volume	200–500	5	Up to compressive strength of 11 MPa	Cured for 28 d in curing chamber, then pre-cracked and immersed in water for 21, 42, 63, 84 and 105 d	- X-ray μCT and SEM/EDS	[81]

**Table 4:** Mortar mixture composition and properties.

Constant Values			
w/c		0.3	
Cementing (kg/m^3^)		550	
Sand/cementing		3	
**Variables Values**			
Mortar age (day)	2	2	28
SCM (% of cement)	0	22%GGBFS + 8%SF	0
f’_c_ (MPa)	24.1	17.0	50.8
E_m_ (GPa)	30	30	39
f_tm_ (MPa)	1.6	1.3	4.0
G_m_ (J/m^2^)	30	20	60

**Table 5:** Capsule and interface properties.

	Variable	Values Used	Range in the Literature	References
Shell geometry	R_s_ (μm)	50, 60, 100	5–1000	[32,33,34,67,71,84,86,87]
	t_s_ (μm)	1, 2, 3, 8	1–200	[32,34,39,53,79,82,84,87,95]
Shell properties	E_s_ (GPa)	4	2.25–12	[32,33,34,39,53,73,77,79,82,84,86,87,96,97,98,99,100,101,102,103]
	f_rs_ (MPa)	30, 50	23–90	[36,84,99,100,101,102,103]
	G_s_ (J/m^2^)	100	40–500	[84,86,97,98,101,102,104,105,106]
	ν_s_	0.3	0.3–0.36	[84,86,87,96,97,98,99,102]
Interface properties	σ_bi_ (MPa)	0.9–3.4	0.1–15	[36,79,82,84,86,87,107,108,109,110,111,112]
	G_i_ (J/m^2^)	20–80	0.1–100	[82,84,86,87]

**Table 6:** Variables considered in the DoE.

Variables	1 Level	2 Levels	3 Levels	Star Point
**2d Mortar without SCM**				
R_s_ (mm)	0.06	0.1		0.05
t_s_ (mm)	0.002	0.003	0.008	0.001
E_s_ (GPa)	4			
f_rs_ (MPa)	30	50		
G_s_ (J/m^2^)	100			
σ_bi_ (MPa)	1.1	1.3		
G_i_ (J/m^2^)	20	50		
**2d Mortar with SCM**				
R_s_ (mm)	0.06	0.1		0.05
t_s_ (mm)	0.002	0.003	0.008	0.001
E_s_ (GPa)	4			
f_rs_ (MPa)	30	50		
G_s_ (J/m^2^)	100			
σ_bi_ (MPa)	0.9	1.0		
G_i_ (J/m^2^)	20	50		
**28d Mortar without SCM**				
R_s_ (mm)	0.06	0.1		0.05
t_s_ (mm)	0.002	0.003	0.008	0.001
E_s_ (GPa)	4			
f_rs_ (MPa)	30	50		
G_s_ (J/m^2^)	100			
σ_bi_ (MPa)	2.9	3.4		
G_i_ (J/m^2^)	40	80		

**Table 7:** Results of the FE.

Run	R_s_ (mm)	t_s_ (mm)	f_rs_ (MPa)	σ_bi_ (MPa)	G_i_ (N/mm)	Failure Mode	CMOD (μm)
1	0.06	0.003	30	1.1	0.02	D	5.187
2	0.10	0.003	30	1.3	0.02	D	5.148
3	0.06	0.008	30	1.1	0.02	D	5.296
4	0.10	0.008	30	1.3	0.02	D	5.145
5	0.06	0.003	50	1.1	0.02	D	5.142
6	0.10	0.003	50	1.3	0.02	D	5.108
7	0.06	0.008	50	1.1	0.02	D	5.258
8	0.10	0.008	50	1.3	0.02	D	5.180
9	0.06	0.003	30	1.1	0.05	D	5.156
10	0.10	0.003	30	1.3	0.05	D	5.121
11	0.06	0.008	30	1.1	0.05	D	5.267
12	0.10	0.008	30	1.3	0.05	D	5.112
13	0.06	0.003	50	1.1	0.05	D	5.170
14	0.10	0.003	50	1.3	0.05	D	5.164
15	0.06	0.008	50	1.1	0.05	D	5.297
16	0.10	0.008	50	1.3	0.05	D	5.147
17	0.06	0.003	30	0.9	0.02	D	4.218
18	0.10	0.003	30	1.0	0.02	D	4.221
19	0.06	0.008	30	0.9	0.02	D	4.371
20	0.10	0.008	30	1.0	0.02	D	4.216
21	0.06	0.003	50	0.9	0.02	D	4.207
22	0.10	0.003	50	1.0	0.02	D	4.188
23	0.06	0.008	50	0.9	0.02	D	4.286
24	0.10	0.008	50	1.0	0.02	D	4.204
25	0.06	0.003	30	0.9	0.05	D	4.192
26	0.10	0.003	30	1.0	0.05	D	4.236
27	0.06	0.008	30	0.9	0.05	D	4.242
28	0.10	0.008	30	1.0	0.05	D	4.193
29	0.06	0.003	50	0.9	0.05	D	4.228
30	0.10	0.003	50	1.0	0.05	D	4.213
31	0.06	0.008	50	0.9	0.05	D	4.230
32	0.10	0.008	50	1.0	0.05	D	4.227
33	0.06	0.003	30	2.9	0.04	D	9.523
34	0.10	0.003	30	3.4	0.04	R	8.132
35	0.06	0.008	30	2.9	0.04	D	9.578
36	0.10	0.008	30	3.4	0.04	D	9.556
37	0.06	0.003	50	2.9	0.04	D	9.629
38	0.10	0.003	50	3.4	0.04	R	9.025
39	0.06	0.008	50	2.9	0.04	D	9.744
40	0.10	0.008	50	3.4	0.04	D	9.515
41	0.06	0.003	30	2.9	0.08	R	9.886
42	0.10	0.003	30	3.4	0.08	R	8.158
43	0.06	0.008	30	2.9	0.08	D	9.614
44	0.10	0.008	30	3.4	0.08	D	9.504
45	0.06	0.003	50	2.9	0.08	D	9.564
46	0.10	0.003	50	3.4	0.08	R	9.056
47	0.06	0.008	50	2.9	0.08	D	9.639
48	0.10	0.008	50	3.4	0.08	D	9.552
49	0.10	0.002	30	1.3	0.02	R	5.115
50	0.10	0.002	50	1.3	0.02	D	5.270
51	0.10	0.002	30	1.3	0.05	R	5.139
52	0.10	0.002	50	1.3	0.05	D	5.202
53	0.10	0.002	30	1.0	0.02	R	4.219
54	0.10	0.002	50	1.0	0.02	D	4.261
55	0.10	0.002	30	1.0	0.05	R	4.167
56	0.10	0.002	50	1.0	0.05	D	4.255
57	0.10	0.002	30	3.4	0.04	R	9.479
58	0.10	0.002	50	3.4	0.04	R	9.726
59	0.10	0.002	30	3.4	0.08	R	9.714
60	0.10	0.002	50	3.4	0.08	R	9.649
61	0.06	0.002	30	1.1	0.02	D	5.170
62	0.06	0.002	50	1.1	0.02	D	5.168
63	0.06	0.002	30	1.1	0.05	D	5.179
64	0.06	0.002	50	1.1	0.05	D	5.161
65	0.06	0.002	30	0.9	0.02	D	4.195
66	0.06	0.002	50	0.9	0.02	D	4.188
67	0.06	0.002	30	0.9	0.05	D	4.267
68	0.06	0.002	50	0.9	0.05	D	4.232
69	0.06	0.002	30	2.9	0.04	R	8.031
70	0.06	0.002	50	2.9	0.04	D	9.602
71	0.06	0.002	30	2.9	0.08	R	8.014
72	0.06	0.002	50	2.9	0.08	D	9.627
73	0.10	0.001	30	1.3	0.02	R	4.546
74	0.10	0.001	50	1.3	0.02	R	5.112
75	0.10	0.001	30	1.3	0.05	R	4.549
76	0.10	0.001	50	1.3	0.05	R	5.208
77	0.10	0.001	30	1.0	0.02	R	3.846
78	0.10	0.001	50	1.0	0.02	R	4.791
79	0.10	0.001	30	1.0	0.05	R	3.838
80	0.10	0.001	50	1.0	0.05	R	4.757
81	0.10	0.001	30	3.4	0.04	R	7.370
82	0.10	0.001	50	3.4	0.04	R	8.111
83	0.10	0.001	30	3.4	0.08	R	7.376
84	0.10	0.001	50	3.4	0.08	R	8.099
85	0.05	0.008	30	1.1	0.02	D	5.189
86	0.05	0.008	50	1.1	0.02	D	5.238
87	0.05	0.008	30	1.1	0.05	D	5.154
88	0.05	0.008	50	1.1	0.05	D	5.179
89	0.05	0.008	30	0.9	0.02	D	4.242
90	0.05	0.008	50	0.9	0.02	D	4.236
91	0.05	0.008	30	0.9	0.05	D	4.219
92	0.05	0.008	50	0.9	0.05	D	4.216
93	0.05	0.008	30	2.9	0.04	D	9.678
94	0.05	0.008	50	2.9	0.04	D	9.597
95	0.05	0.008	30	2.9	0.08	D	9.728
96	0.05	0.008	50	2.9	0.08	D	9.785
